# The Value Placed on Expert Testimony by an Atlanta Jury

**Published:** 1885-04

**Authors:** 


### Editorial.

### THE VALUE PLACED ON EXPERT TESTIMONY BY AN ATLANTA JURY.

The status of professional experts in any kind of medico-legal investigation is a matter of great interest to all members of the medical profession, and the attitude of this class of witnesses in cases of lunacy becomes highly important likewise to the community in which such inquiry is instituted. The public sentiment of this country has been awakened recently to the consequences attached to a decision as to the value of professional testimony compared with other sources of information in the Rhilander case. It is generally known that Mr. William C. Rhilander committed a homicidal assault upon Mr. John Drake, and being brought to trial, a commission was appointed by Recorder Smyth, consisting of Dr. William Detmold, Mr. Patrick Nolan and Mr. Edward Patterson. After a large amount of evidence was taken a report was made to the court, in which two of the commissioners, Dr. Detmold and Mr. Nolan, gave the opinion that the prisoner was insane, while Mr. Patterson gave a contrary opinion. Recorder Smyth has recently, after reviewing the evidence furnished by the commission, reversed its decision and decided that the defendant is sane. In this case the weight of scientific opinion was almost entirely on the side of Rhilander's insanity ; and the Journal of Insanity, from which we gather these facts, states that the case, besides furnishing a precedent that a judge may reverse the decision of a commission he appoints, is instructive, in that it shows how valueless expert testimony may still be, even under the law. The issue is fully made here between the matured opinion of a qualified scientific witness and the judgment of a court based upon outside evidence, so that it becomes the medical profession to scrutinize closely the tendency of such a decision.

But it is not only in such a case as this, in which the alleged insanity is denied as a palliation of the otherwise criminal act of the individual, that we are called upon to consider the bearings of such proceeding, as an instance has occurred in the Ordinary's Court of Atlanta in which insanity was decreed and a commitment to a lunatic asylum made out, with provision of a guardian, for an individual who was pronounced sane by the medical witness in the case.

The wife consults the physicians in charge as to the advisability of sending the man to the asylum while suffering from the effects of apoplexy. She is advised against it, but seeks the ordinary and swears out a writ of lunacy, having the husband tried and introducing neighbors as witnesses secures his commitment.

The verdict was given by the jury against the testimony of the physician, made under oath to the court. The evidence of a neighbor who was not a physician outweighed the evidence in the decision of the case by the jury.

The State Asylum being full, so that the patient could not be admitted, he is put under the care of a guardian and his effects placed at his disposition. In the meantime he recovers from the disease to find himself adjudged a lunatic, and is subjected to all the vexations and embarrassments growing out of such proceedings.

It does not affect the exception taken to such action that "any person for whom a guardian is appointed under this article (of the Code) upon restoration to sanity and capacity, may personally or by attorney petition the ordinary setting forth the fact and praying the revocation of such guardianship," as the terms and specifications are objectionable. "Upon such petition the Ordinary may examine into the truth thereof; and if satisfied of its truth, and the guardian consenting thereto, the Ordinary shall grant the prayer and order the guardian forthwith to deliver over to such person his property, money and effects." It is evident that should there be any motive of self-interest on the part of said guardian, or of those obtaining his appointment, his consent will not be given, and hence the wrong done is not redressed until the Ordinary is satisfied of the truth of the petition and institutes further proceedings.

All in all, the question turns upon the competency of the witnesses upon whose testimony the commitment for lunacy is made out, or the appointment of a guardian is decreed, and the issue rests upon the validity or not of the opinion of a medical expert against that of unqualified persons. It is granted that every medical man who may be called up to give evidence in a trial of this kind is not to be recognized as a competent expert in questions of lunacy, yet allowing the claim of good faith and honesty, his opinion should be entitled to more consideration than those entirely unacquainted with physical and mental disorders.

